# Epinephrine responsiveness is reduced in livers from trained mice

**DOI:** 10.14814/phy2.14370

**Published:** 2020-02-15

**Authors:** Hana A. Dibe, Logan K. Townsend, Greg L. McKie, David C. Wright

**Affiliations:** ^1^ Department of Human Health and Nutritional Sciences University of Guelph Guelph ON Canada

**Keywords:** epinephrine, glycogen, liver, Mice

## Abstract

The liver is the primary metabolic organ involved in the endogenous production of glucose through glycogenolysis and gluconeogenesis. Hepatic glucose production (HGP) is increased via neural‐hormonal mechanisms such as increases in catecholamines. To date, the effects of prior exercise training on the hepatic response to epinephrine have not been fully elucidated. To examine the role of epinephrine signaling on indices of HGP in trained mice, male C57BL/6 mice were either subjected to 12 days of voluntary wheel running or remained sedentary. Epinephrine, or vehicle control, was injected intraperitoneally on day 12 prior to sacrifice with blood glucose being measured 15 min postinjection. Epinephrine caused a larger glucose response in sedentary mice and this was paralleled by a greater reduction in liver glycogen in sedentary compared to trained mice. There was a main effect of epinephrine to increase the phosphorylation of protein kinase‐A (p‐PKA) substrates in the liver, which was driven by increases in the sedentary, but not trained, mice. Similarly, epinephrine‐induced increases in the mRNA expression of hepatic adrenergic receptors (*Adra1/2a, Adrb1*), and glucose‐6‐phosphatase (*G6pc*) were greater in sedentary compared to trained mice. The mRNA expression of cAMP‐degrading enzymes phosphodiesterase 3B and 4B (*Pde3b, Pde4b)* was greater in trained compared to sedentary mice. Taken together, our data suggest that prior exercise training reduces the liver's response to epinephrine. This could be beneficial in the context of training‐induced glycogen sparing during exercise.

## Graphical abstract

We have found that prior exercise training blunts the ability of epinephrine to increase blood glucose and reduce liver glycogen. This training adaptation could have relevance to our understanding of fuel partitioning.

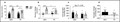

## 1 INTRODUCTION

Exercise‐induced activation of the sympathetic nervous system results in increased production and secretion of the catecholamines epinephrine and norepinephrine, from the adrenal medulla and postganglionic neurons, respectfully (Leosco et al., 2013). Plasma catecholamine levels increase at the onset of exercise and continue to rise in an intensity and duration‐dependent manner (Howlett et al., 1999). Norepinephrine and epinephrine bind to G‐protein coupled beta adrenergic receptors (β1, β2, β3), albeit with different affinities (Molinoff, 1984), to facilitate the physical interaction between the receptor and its stimulatory protein subunit (i.e., G_s_α) leading to a conformational change in G_s_α that activates adenylyl cyclase. Adenylyl cyclase propagates the signal downstream by increasing cytosolic levels of 3′,5′‐cyclic AMP (cAMP), which subsequently activates protein kinase A (PKA) (Erraji‐Benchekroun et al., 2005; Pierce, Premont, & Lefkowitz, 2002). When activated, PKA can phosphorylate numerous enzymes, regulating a variety of metabolic pathways throughout the body. In skeletal muscle and liver, the activation of PKA increases glycogen breakdown (Jensen, Brennesvik, Lai, & Shepherd, 2007; Johanns et al., 2016). In the liver, PKA also stimulates gluconeogenesis through the transcriptional activation of numerous genes, including glucose‐6‐phosphatase (*G6pc*) and phosphoenolpyruvate carboxykinase (*Pepck*) (Xie et al., 2018). In adipose tissue, increases in PKA activity stimulates lipolysis through the phosphorylation of perilipin proteins and multiple lipases such as hormone sensitive lipase (Anthonsen, Rönnstrand, Wernstedt, Degerman, & Holm, 1998; Strålfors et al., 1984). In the pancreas, epinephrine stimulates glucagon secretion from the α‐cells of the pancreas, and inhibits insulin release from β‐cells (Hamilton et al., 2018; Han & Bonen, 1998; Peterhoff et al., 2003). cAMP‐PKA signaling is attenuated by cyclic nucleotide phosphodiesterases (PDE), which can be activated in response to various cellular signals including protein kinase B (AKT) and 5′AMP‐activated protein kinase (AMPK) (Berger et al., 2009; Johanns et al., 2016).

Exercise training influences the metabolic responsiveness to catecholamines in adipose tissue, skeletal muscle, and the liver (Plourde, Rousseau‐Migneron, & Nadeau, 1993; Podolin, Gleeson, & Mazzeo, 1996; Riviere, Crampes, Beauville, & Garrigues, 1989; Sumida, Arimoto, Catanzaro, & Frisch, 2003). For example, isoproterenol‐stimulated adenylyl cyclase activity was significantly elevated in soleus muscles from trained compared to sedentary rats (Plourde et al., 1993). In trained subjects, the lipolytic response of adipocytes to epinephrine was increased in comparison to fat cells from sedentary subjects (Riviere et al., 1989). Similarly, epinephrine stimulated gluconeogenesis was greater in hepatocytes isolated from trained compared to sedentary rats (Sumida et al., 2003), though it should be noted that this has not been a universal finding (Podolin et al., 1996).

Exercise training confers a glycogen sparing effect, such that reductions in liver glycogen during exercise are attenuated in trained compared to sedentary subjects (Baldwin, Fitts, Booth, Winder, & Holloszy, 1975; Fitts, Booth, Winder, & Holloszy, 1975; Gonzalez, Fuchs, Betts, & Loon, 2016). The specific cellular mechanisms mediating this effect are not known but it is tempting to speculate that alterations in the ability of epinephrine to stimulate hepatic glycogenolysis could be involved. Within this framework, the purpose of the present investigation was to examine the impact of exercise training on epinephrine‐induced increases of indices of PKA signaling and glycogenolysis in liver. We hypothesized that exercise training would attenuate hepatic responsiveness to epinephrine, as shown by attenuated increases in PKA signaling, liver glycogen breakdown, and blood glucose.

## 2 METHODS

### 2.1 Animals and ethics

Eight‐week‐old male C57BL/6 mice (Charles River) were individually housed at room temperature (~22°C) and kept on a 12:12 hr light‐dark cycle (08:00–20:00 hr). Mice were given one week to acclimate to their environment before experiments began. Mice had ad libitum access to a standard rodent diet (cat no. 7004; Teklad) and water. All cages contained standard corncob bedding and a tube for environmental enrichment. All animal protocols were approved by the University of Guelph Animal Care Committee and followed the Canadian Council on Animal Care (CCAC) guidelines. A total of 108 mice were used for the experiments described below.

### 2.2 12‐day exercise intervention

Following a one‐week acclimation period, mice were weight‐matched into sedentary (SED) and exercise trained (TR) groups (*n* = 10/group). SED mice remained individually housed in standard shoebox cages while TR mice were moved to individual cages with running wheels and wired bike computers (M3.1 WR VDO Cycle Computing) to track distance and speed. TR mice had access to wheels for 12 days, a length chosen based off previous work that demonstrated significant metabolic adaptations with this 12‐day voluntary wheel running (VWR) protocol (Knuth et al., 2018). Food intake, body weight, running distance and running time were recorded every other day for all mice.

### 2.3 Glucose tolerance test

On day 10 of the 12‐day exercise intervention, a subset of mice from each group (*n* = 10) were subject to an intraperitoneal (i.p.) glucose tolerance test (GTT) at ~15:00 hr. SED and TR mice were fasted for 4 hr prior to the GTT with a wheel lock in place for the TR mice overnight so as to avoid any residual effects of the last bout of exercise (Knuth et al., 2018). Mice were injected i.p. with a weight‐adjusted bolus of D‐glucose (2 g/kg BW). Blood was sampled from a tail snip and glucose measured with a handheld glucometer (Freestyle Lite, 70 Abbott Laboratories) immediately preinjection (time point 0) and at 15, 30, 60, 90, and 120 min postinjection.

### 2.4 Epinephrine challenge and tissue collection

Norepinephrine is 30‐fold less effective in stimulating HGP than epinephrine, and thus plays a minor role in glucose regulation compared to epinephrine (Coker, Krishna, Lacy, Bracy, & Wasserman, 1997; Connolly et al., 1991; Sacca, Morrone, Cicala, Corso, & Ungaro, 1980); therefore, we chose to focus solely on epinephrine in this study. Thus, an epinephrine dose–response experiment was first conducted in vivo to determine the lowest dosage that would stimulate a significant increase in HGP. Mice were injected i.p. with epinephrine or vehicle at a dose of 0.5, 0.25, or 0.125 mg/kg BW, and blood glucose was measured at baseline and at 15 min postinjection. We chose this time point in an effort to minimize compensatory neuroendocrine changes brought about by prolonged increases in blood glucose. At 15 min, blood glucose concentrations were significantly elevated in the 0.5 mg/kg BW group only (*p* < .05; Table 1). As such, this dose and time point were chosen for use in subsequent experiments. On day 12 of the 12‐day exercise intervention, mice were weight and distance matched into groups receiving an i.p. injection of epinephrine (EPI) (0.5 mg/kg BW) or vehicle (VEH). TR mice had wheels locked 4 hr before injection. Mice had access to food during the experiment which began at 13:00 hr. Blood glucose was measured at baseline and 15 min postinjection. After 15 min, mice were anesthetized with sodium pentobarbital (∼5 mg/100 BW) and blood collected via cardiac exsanguination. Liver and triceps were collected, immediately frozen in liquid nitrogen and stored at −80°C. Blood was allowed to clot at room temperature and centrifuged at 2,000 *g* for 10 min before serum was aliquoted and stored at −80°C.

**Table 1:** Blood glucose 15 min after epinephrine injection of varying doses

	Epinephrine dose (mg/kg BW)			
	VEH	0.125	0.25	0.5
Blood glucose	8.86 ± 0.67	10.38 ± 0.71	10.24 ± 1.54	13.16 ± 1.131*

Note

Data presented as mean ± SEM (*n* = 5 mice/group).

Abbreviation: VEH, vehicle.

* Denotes significant (*p* < .05) difference from VEH.

### 2.5 Acute voluntary wheel running

To determine if a single bout of exercise could affect liver responsiveness to epinephrine, we utilized a single overnight VWR model. Following a one‐week acclimation period, mice (*n* = 7) were weight matched into sedentary (SED) and exercised (EX) groups, and cages set up as described above. Mice had access to wheels from 16:00 to 08:00 hr the following day. Body weights, food intake, and distance ran were recorded after the night of running. Wheels were locked at 08:00 hr and mice were subject to epinephrine or vehicle injection as described above. Blood glucose was measured at baseline and 15 min postinjection with tissues collected as previously described.

### 2.6 Serum measurements

Nonesterified fatty acids (NEFA; Wako Diagnostics) and glycerol (cat no. F6428; Sigma Aldrich) were measured on a 96‐well plate as previously described (MacPherson, Castellani, Beaudoin, & Wright, 2014). Glucagon and insulin were measured via enzyme‐linked immunosorbent assays (ELISA) (cat no. 10‐1281‐01; 10‐1247‐013; Mercodia). All assays were conducted in accordance with the manufacturer's instructions.

### 2.7 Hepatic glycogen content

Livers from all mice were freeze‐dried and dissected free of visible blood and contaminating hair for glycogen analysis. Glycogen content was determined from an 0.1–2.0 mg aliquot of freeze‐dried liver (McKie et al., 2019). Glucose and hexose monophosphates were degraded using 0.1 M NaOH incubated in an 80°C water bath for 10 min. Samples were then neutralized in a buffer containing 0.1 M HCl, 0.2 M citric acid, and 0.2 M Na_2_HPO_4_· H_2_O. Glycogen was enzymatically hydrolyzed to glucose with amyloglucosidase (cat no. A7095; Sigma Aldrich) and incubated for 1 hr at room temperature. Glucose was measured directly by fluorometric assay using equal volumes of hexokinase (cat no. H‐4502; Sigma Aldrich) and glucose‐6‐phosphate dehydrogenase (cat no. G‐5885; Sigma Aldrich) (Liang, Donthi, Kralik, & Epstein, 2002). Glycogen concentrations were calculated from the molar absorptivity of NADPH (340 nm = 6.22 cm^2^/mol at 37°C) and expressed as mmol/kg dry weight.

### 2.8 Western blotting

Tissues were homogenized in 30x or 20x (liver or tricep tissue weight, respectively) cell lysis buffer (cat no. FNN0021; ThermoFisher) containing phenylmethylsulfonyl, and protease inhibitor (Sigma Aldrich). Following this, samples were centrifuged (4°C for 10 min at 5,000 *g*) and the supernatant collected. A bicinchoninic acid assay was used to determine protein content in order to prepare samples to a final concentration of 1 ug/ul (Smith et al., 1985). Samples were prepared with cell lysis buffer and 2x laemmli sample buffer. Samples were loaded in equal amounts, separated on 15‐well, 10% or 5% SDS‐polyacrylamide gels, and transferred onto a nitrocellulose membrane. Membranes were blocked with nonfat skim milk powder diluted (5%) in TBST for 1 hr and incubated overnight at 4°C with primary antibody (diluted 1:1,000). The following day, membranes were incubated with the corresponding secondary antibody (anti‐rabbit or anti‐mouse horseradish peroxidase‐conjugated antibody) for 1 hr at room temperature. ECL substrate (cat no. 1705061; Bio‐Rad Laboratories) was used to visualize bands through chemiluminescence on a FluorChem FC2 machine and protein content was quantified by densitometry using AlphaView Software (Cell Biosciences). Ponceau S (cat no. P7170; Sigma Aldrich) staining or a housekeeping protein (Vinculin or GAPDH) were used as a loading control (Townsend et al., 2019). Phosphorylated and total protein were expressed relative to their within‐gel loading control, then phosphorylated proteins were normalized to total protein (i.e., phosphor/total).

Primary antibodies were obtained from Cell Signaling (Danvers, MA, USA) for protein kinase B total (Akt, cat no. 9272), phosphorylated protein kinase b (pAkt^Ser473^, cat no.9271; pAkt^Thr308^, cat no. 9275), acetyl‐CoA carboxylase total (ACC, cat no. 3676), phosphorylated acetyl‐CoA carboxylase (pACC ^Ser79^, cat no. 3661), AMPKα (cat no. 2532), phosphorylated‐AMPKα Thr172 (pAMPKα, cat no. 2535), phosphorylated protein kinase A substrates (pPKA‐s, cat no. 9624), cytochrome c (CYTO C, cat no. 4272), hexokinase II (HKII, cat no. 2106); from Abcam for ubiquinol‐cytochrome C reductase (CORE1, cat no. 110252), cytochrome c oxidase subunit IV (COX IV, cat no. 16056), peroxisome proliferator‐activated receptor‐γ coactivator‐1α (PGC1α, cat no. 54481), citrate synthetase (CS, cat no. 129095); from Cayman Chemicals for phosphoenolpyruvate carboxykinase (PEPCK, cat no. 10004943); from Santa Cruz Biotechnology for glucose‐6‐phosphatase (G6Pase, cat no. 25840) and from Origene for phosphodiesterase 4B (PDE4B, cat no. TA503471).

### 2.9 Real‐time quantitative PCR

Liver (30–40 mg) was homogenized in 1 ml of TRIzol (cat no. 15596018; ThermoFisher Scientific) and RNA was extracted using Bio Basic EZ‐10 Spin Column Total RNA Miniprep Super Kits (cat no. BS784; Bio Basic) as described previously (Frendo‐Cumbo et al., 2016; Peppler, Townsend, Knuth, Foster, & Wright, 2018; Townsend et al., 2019). Synthesis of cDNA was completed using Superscript II (cat no. 4368814; ThermoFisher Scientific) and reverse transcription quantitative PCR was run using SYBR Green Supermix (cat no. 1725271; Bio‐Rad Laboratories) using PCR primers (Table 2) on a Bio‐Rad CFX Connect system. All values are expressed relative to the housekeeping gene *Ppib* and normalized to the SED VEH group using the 2^−∆∆^ *^Ct^* method (Livak & Schmittgen, 2001). Expression of the housekeeping gene was stable and did not change with intervention.

**Table 2:** mRNA primers

Gene (Ref)	Primer Sequence, 5′–3′	
	Forward	Reverse
*Adra1a* (Spandidos et al., 2009)	AGTGGGTGTCTTCCTAGCC	GCCTAGAACCTCCATAGTGGC
*Adra2a* (Spandidos et al., 2009)	GGTGACACTGACGCTGGTTT	ACTGGTGAACACCGCGATAATA
*Adrb1* (Hauck et al., 2017)	CTCATCGTGGTGGGTAACGTG	ACACACAGCACATCTACCGAA
*Adrb2* (Hauck et al., 2017)	GGGAACGACAGCGACTTCTT	GCCAGGACGATAACCGACAT
*G6pc* (Gray et al., 2015)	AGGAACGCCTTCTATGTCCTCTTTC	GCGTTGTCCAAACAGAATCCACTTG
*Pck1* (Gray et al., 2015)	CGGAAGAGGACTTTGAGAAAGCTTC	GCGAGTCTGTCAGTTCAATACCAATC
*Pde3b* (Niiya et al., 2001)	TGAGTGGCAGAACCAGTTTCC	TGCGATCCCACCTTGAACA
*Pde4b* (Avila et al., 2016)	GACCGGATACAGGTTCTTCG	CAGTGGATGGACAATGTAGTCA
*Ppib* (Spandidos et al., 2009)	GGAGATGGCACAGGAGGAA	GCCCGTAGTGCTTCAGCTT

### 2.10 Statistical analyses

**Figure 1:**
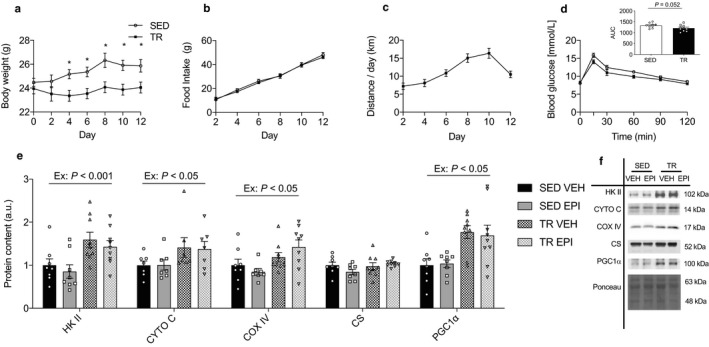
12 days of voluntary wheel running attenuated weight gain despite similar food intake and increased markers of mitochondrial content in triceps muscles. Body weight (a) and food intake (b) was measured every other day over a 12‐day period in SED and TR mice (*n* = 10). Average VWR distance recorded every two days in TR mice (*n* = 10) (c). Glucose tolerance test (*n* = 10) conducted on the 10th day of VWR, with inset showing AUC for entire 120 min (d). Quantified western blots for triceps protein content of HKII, CYTO C, COX IV, CS, PGC1α (e) and representative western blot images (f) (*n* = 8–9), as well as a representative ponceau image. Average body weight was analyzed by multiple *t* tests using the Holm–Sidak Method. AUC was analyzed by an unpaired *t* test. *denotes statistical significance (*p* < .05) between groups. Triceps protein content was analyzed by two‐way ANOVA. Ex—denotes main effect of exercise training. Epi—denotes main effect of epinephrine. Individual data points are shown where possible. All data are presented as mean ± SEM

Data were screened for outliers using the Extreme Studentized Deviate (ESD) method. This method is used to detect outliers in univariate data sets with approximately normal distribution. Identified outliers were not included in data analyses. Normality of residuals was assessed using the Shapiro–Wilk test. Data in Figure 1a‐cytochrome C, 3*D*, 4*D*, 6*A*‐pAKT S473, and 7*C* were log_10_ transformed as they did not pass the test (*p ≤ *.05). Data were analyzed using either an unpaired student's *t* test (e.g., average body weight change and delta liver glycogen) or a two‐way analysis of variance (ANOVA; e.g., to test the effects of, or interactions between, exercise and epinephrine). Post hoc tests using Tukey's multiple comparisons test were performed when significant interactions were reported. Statistical analyses and graphs were made using Prism 6.0 (GraphPad Software). Significance was set at an alpha level of 0.05 and data is displayed as the mean ± SEM with individual data points shown when possible.

## 3 RESULTS

### 3.1 12 days of VWR exercise training attenuates weight gain despite similar food intake

We first wanted to verify the efficacy of our 12‐day exercise training model. Average body weight of TR mice did not change throughout the intervention while SED mice gained an average of 1.5 g (*p* < .05; Figure 1a). There were no differences in food intake (*p* = .36) between groups (Figure 1b). There were no differences in running distance and time between TR‐VEH and TR‐EPI groups. TR‐VEH and TR‐EPI mice ran an average of 60.5 ± 7.6 km and 57.6 ± 6.1 km for a total average of 70.6 ± 4.2 hr and 69.8 ± 1.0 hr, respectively. Results of the GTT (Figure 1d) indicated a nonsignificant trend (*p* = .052) in glucose tolerance between groups. Exercise is well known to increase skeletal muscle oxidative capacity (Knuth et al., 2018; McKie et al., 2019; Peppler et al., 2018; Stanford, Middelbeek, & Goodyear, 2015) and in the current study, we observed consistent exercise‐induced increases in the protein content of HKII (*p* < .001), CYTO C, COX IV, and PGC1α (*p* < .05; Figure 1e) in the triceps muscle.

### 3.2 Exercise training attenuates the increase in blood glucose in response to epinephrine

**Figure 2:**
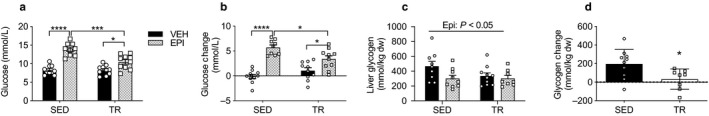
The acute blood glucose response to an epinephrine injection is blunted by exercise training. VWR attenuated the blood glucose response at 15 min after an epinephrine injection (0.5 mg/kg BW) (a; *n* = 10). The change in blood glucose from baseline (*t* = 0) (b). Hepatic glycogen content (c; *n* = 10) and the relative decline in glycogen between SED and TR mice after epinephrine administration (d). Data analyzed by two‐way ANOVA. Epi—denotes main effect of epinephrine. Significant interactions analyzed by post‐hoc comparison denoted by *p < .05, ***p < .001, ****p < .0001. Individual data points are shown where possible. All data are presented as mean ± SEM

After characterizing our 12‐day exercise training model, we sought to determine if exercise training impacted acute epinephrine‐induced increases in blood glucose. SED EPI mice had a significantly larger spike in blood glucose 15‐min postepinephrine administration compared to TR EPI mice (*p* < .05; Figure 2a). Additionally, the change in glucose from baseline (*t* = 0) to 15‐min postepinephrine (*t* = 15), was greater in SED‐EPI compared to TR‐EPI mice (*p* < .05; Figure 2b). Epinephrine is well known to decrease liver glycogen content, however, we wanted to investigate if there was an exercise training adaptation to attenuate epinephrine‐induced glycogen depletion. Interestingly, as little as 15 min after epinephrine administration, there was a reduction in hepatic glycogen content in SED and TR mice (*p* < .05; Figure 2c). The relative change in glycogen content following epinephrine administration was calculated by subtracting the individual glycogen values of the epinephrine‐treated groups from the average value of the vehicle control group. In doing so, the relative decline in glycogen in EPI compared to VEH controls, was larger in SED than TR mice (*p* < .05; Figure 2d).

### 3.3 Exercise training does not impact the effect of epinephrine on circulating markers of lipolysis and pancreatic hormones

**Figure 3:**
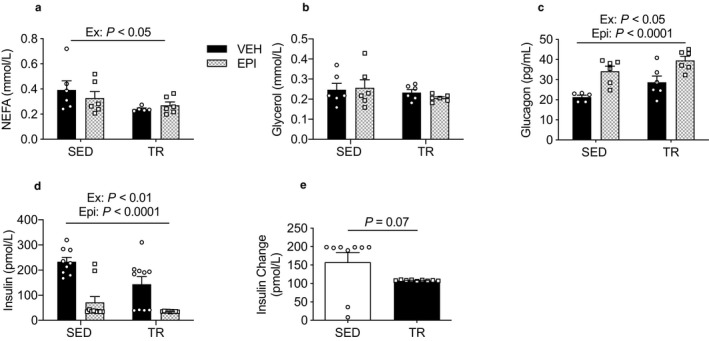
Circulating metabolites and glucoregulatory hormones are altered in response to an acute epinephrine injection. Serum NEFA (a), glycerol (b), glucagon (c), and insulin (d) concentrations and the relative change in insulin (e) following termination (*n* = 6–10). Data were analyzed by two‐way ANOVA. Ex—denotes main effect of exercise training. Epi—denotes main effect of epinephrine. Individual data points are shown where possible. All data are presented as mean ± SEM

Previous studies have demonstrated that exercise training enhances catecholamine‐stimulated lipolysis (Kawai, 1983; Pierce et al., 2002). To determine if exercise impacted indices of lipolysis following acute epinephrine exposure, we measured serum NEFA and glycerol 15 min postepinephrine or vehicle injections. There was a main effect of exercise to reduce circulating NEFA concentrations (*p* < .05), despite circulating glycerol concentrations being similar between groups (*p* = .25; Figure 3a,b).

In addition to having direct effects on the liver, epinephrine increases glucagon while reducing serum insulin concentrations (Hamilton et al., 2018; Sherwin & Sacca, 1984; Zhang, Wan, Liu, Hua, & Sun, 2018). As insulin suppresses, and glucagon increases, hepatic glycogenolysis and hepatic gluconeogenesis (Heyworth, Wallace, & Houslay, 1983; Petersen, Laurent, Rothman, Cline, & Shulman, 1998; Wasserman et al., 1989), we wanted to determine if the blunted epinephrine‐mediated increase in blood glucose and liver glycogenolysis in exercised mice could be related to differences in these hormones. There was a main effect of both epinephrine (*p* < .0001) and exercise (*p* < .05) to increase circulating glucagon levels (Figure 3c). There was a main effect of epinephrine (*p* < .0001) and exercise (*p* < .001) (Figure 3d) to reduce serum insulin, with a nonsignificant trend (*p* = .07; Figure 3e) for the relative decline in insulin with epinephrine treatment to be larger in SED mice. Taken together, these findings provide evidence that epinephrine‐induced changes in insulin and glucagon do not explain the blunted reduction in liver glycogen and blunted rise in blood glucose in TR mice.

### 3.4 Exercise training reduces epinephrine‐induced increases in gluconeogenic gene expression

**Figure 4:**
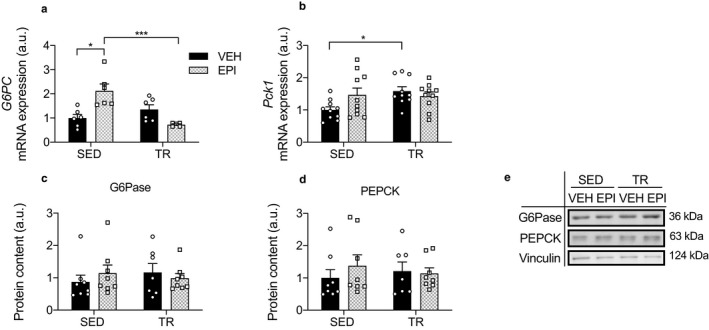
Hepatic gene expression and protein content of gluconeogenic markers. Relative hepatic mRNA expression of *G6pc* (a) (*n* = 6) and *PCK1* (b) (*n* = 10). Protein content of G6Pase (c) and PEPCK (d) (*n* = 7–8) and representative Western blot images (e). Data were analyzed by two‐way ANOVA. Significant interactions analyzed by post‐hoc comparison denoted by *p < .05, ***p < .001. Individual data points are shown where possible. All data are presented as mean ± SEM

It is well known that epinephrine activates gluconeogenic machinery in the liver (Sherwin & Sacca, 1984; Sokal & Sarcione, 1959). To test if the blunted increase in glucose output in trained mice was due to a downregulation of gluconeogenic enzymes, we measured G6Pase and PEPCK gene expression and protein content. There was an exercise by epinephrine interaction for *G6pc* expression such that epinephrine increased mRNA expression by 212% in SED mice, compared to a 46% decrease in TR mice (*p* < .001; Figure 4a). There was an exercise by epinephrine interaction for *Pck1* mRNA expression (*p* = .046). Specifically, exercise increased expression in VEH mice by 59% (*p* < .05; Figure 4b). However, there were no differences in the protein content of G6Pase and PEPCK across groups (Figure 4c,d).

### 3.5 Epinephrine alters mRNA expression of α‐ and β‐adrenergic receptors

**Figure 5:**
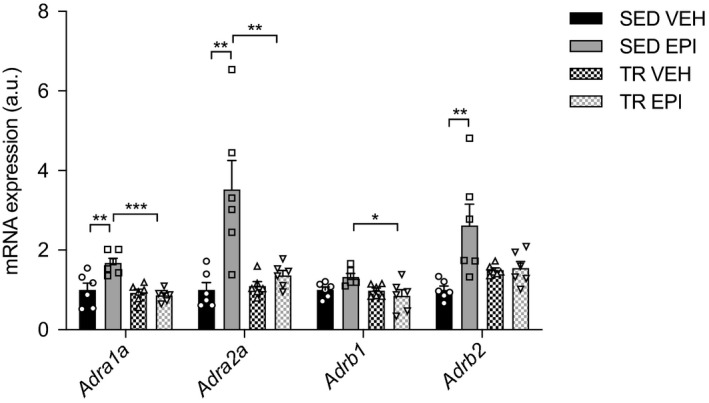
Epinephrine increases gene expression of adrenergic receptors in the liver. Relative hepatic mRNA expression of *Adra1a*, *Adra2a*, *Adrb1*, and *Adrb2* adrenergic receptors (*n* = 6). Data were analyzed by two‐way ANOVA. Significant interactions analyzed by post hoc comparison denoted by *p < .05, **p < .01, ***p < .001. Individual data points are shown where possible. All data are presented as mean ± SEM

In order to examine if exercise impacts adrenergic receptor content, we measured α‐ and β‐adrenergic receptor gene expression (*Adra1a, Adra2a*, *Adrb1* and *Adrb2*) in the liver. Epinephrine increased *Adra1a* (*p* < .001)*, Adra2a* (*p* < .01) *and Adrb1* (*p* < .05) expression in SED, but not TR mice (Figure 5). There was an exercise by epinephrine interaction for *Adrb2* expression (*p* = .0123) such that epinephrine increased expression in SED mice (*p* < .01) while this effect was absent in TR mice (Figure 5). These data suggest that exercise alters the adrenergic receptor response to an epinephrine challenge and provides further evidence of a reduced responsiveness to epinephrine in the liver with exercise training.

### 3.6 Epinephrine activates PKA and reduces insulin signaling in the liver from both sedentary and trained mice

**Figure 6:**
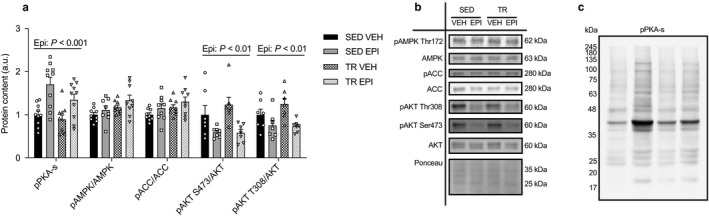
Epinephrine activates PKA and reduces markers of insulin signaling in the livers of SED and TR mice. Hepatic protein content (a) and representative western blot images (b) for pPKA‐s, pAMPK/AMPK, pACC/ACC, pAKT S473/AKT, and pAKT T308/AKT (*n* = 9–10). A representative image for phospho‐PKA substrate blot (c). Data were analyzed by two‐way ANOVA. Ex—denotes main effect of exercise training. Epi—denotes main effect of epinephrine. Individual data points are shown where possible. All data are presented as mean ± SEM

Next, we aimed to determine whether the blunted response to epinephrine was reflected by changes in PKA signaling, and reputed negative regulators of PKA, including AMPK and AKT. The activation of PKA is known to increase glycogenolysis and gluconeogenesis and the phosphorylation status of PKA substrates can be used as an indicator of downstream PKA activity (Mowers et al., 2013). There was a main effect of epinephrine to increase the phosphorylation of PKA substrates in the livers of SED and TR mice (*p* < .001) (Figure 6a), with post hoc analysis revealing that the main effect of epinephrine was driven by significant increases in SED (*p* < .01) but not TR mice. AMPK acts as a cellular energy sensor that regulates glucose and lipid metabolism and can interfere with PKA signaling in hepatocytes (Andreelli et al., 2006; Johanns et al., 2016; Steinberg & Carling, 2019). There were no differences in the phosphorylation of AMPK or the downstream target ACC (Figure 6a). The activation of AKT has been shown to attenuate PKA signaling (Kitamura et al., 1999); As shown in Figure 6, and consistent with the circulating insulin data, there was a main effect of epinephrine to reduce the phosphorylation of AKT on both threonine and serine activation sites (pAKT‐Thr308, *p* < .01; pAKT‐Ser473; *p* < .01). Together these finding provide evidence that differences in AKT and AMPK signaling do not explain the protective effects of exercise training against epinephrine‐induced increases in blood glucose and liver glycogen breakdown.

### 3.7 PDE3B and PDE4B in liver are increased with exercise training

**Figure 7:**
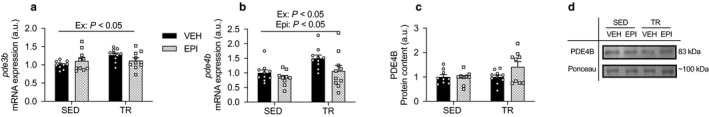
Changes in cAMP‐degrading enzyme phosphodiesterase 3B and 4B do not account for altered glucose responses between SED and TR mice. Relative hepatic mRNA expression of PDE3B (a) and PDE4B (b) (*n* = 10). Hepatic protein content (c) and representative western blot images (d) for PDE4B (*n* = 10). Data were analyzed by two‐way ANOVA. Ex—denotes main effect of exercise training. Epi—denotes main effect of epinephrine. Individual data points are shown where possible. All data are presented as mean ± SEM

Epinephrine increases hepatic glycogenolysis and HGP in a cAMP‐PKA‐dependent pathway (Bousquet‐Mélou, Galitzky, Moreno, Berlan, & Lafontan, 1995; Kolnes et al., 2015; Wasserman & Cherrington, 1991). Phosphodiesterase 3B and 4B (PDE3B, PDE4B) are the primary enzymes responsible for the degradation of cAMP in the liver (Avila, Barker, Zhang, McClain, & Barve, 2016; Berger et al., 2009; Omori & Kotera, 2007; Wahlang, McClain, Barve, & Gobejishvili, 2018). Therefore, in order to determine if epinephrine responsiveness was associated with changes in phosphodiesterase expression, we measured *Pde3b* and *Pde4b* gene expression in the liver. There was a main effect of exercise to increase *Pde3b* and *Pde4b* expression (*p* < .05, *p* < .01; Figure 7a,b), and a main effect of epinephrine to decrease *Pde4b* expression (*p* < .05) (Figure 7b). PDE4B protein content was unaffected by exercise or epinephrine (Figure 7c). Unfortunately, we could not determine PDE3B protein content as we could not obtain a quality primary antibody.

### 3.8 Acute VWR does not induce liver epinephrine resistance

**Figure 8:**
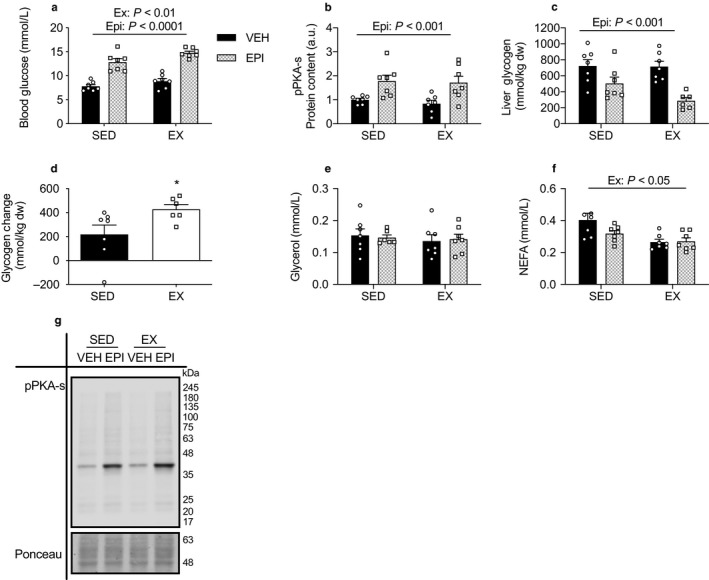
Acute VWR does not change the metabolic response to epinephrine. Blood glucose concentration 15 min after epinephrine injection (a). Hepatic protein content (b) for pPKA‐s (*n* = 7). Hepatic glycogen content (c; *n* = 10) and the relative decline in glycogen between SED and EX mice after epinephrine administration (d). Glycerol (e) and NEFA (f) plasma concentrations calculated following termination (*n* = 7). Representative Western blot images for p‐PKA‐s (g). Data were analyzed by two‐way ANOVA. Ex—denotes main effect of acute VWR. Epi—denotes main effect of epinephrine. *p < .05. Individual data points are shown where possible. All data are presented as mean ± SEM

A single bout of exercise can have long‐lasting metabolic effects (Richter, Mikines, Galbo, & Kiens, 1926; Steenberg et al., 2019; Sylow et al., 2019). Therefore, to determine if a single night of wheel running could affect liver responsiveness to epinephrine, the previous experiment was repeated, however, exercised mice (EX) ran on wheels for only one night (16:00–08:00 hr) instead of 12 days. EX mice ran an average of 4.07 ± 0.58 km for a total average time of 7.54 ± 0.73 hr. There was a main effect of epinephrine (*p* < .0001) and overnight VWR (*p* < .01) to increase blood glucose (Figure 8a). Liver protein content of pPKA substrates was increased with epinephrine treatment with no effect of exercise (*p* < .001, Figure 8b,g). Epinephrine reduced hepatic glycogen content (*p* < .001) and EX mice experienced a greater depletion in hepatic glycogen levels (*p* < .05, Figure 8c,d). Epinephrine and exercise did not impact serum glycerol concentrations (Figure 8e), however there was a main effect of overnight VWR to reduce serum NEFA concentrations (*p* < .05; Figure 8f). Thus, a single bout of exercise does not alter the hepatic response to an epinephrine challenge.

## 4 DISCUSSION

As epinephrine plays a central role in fuel mobilization during exercise and given the large contribution of the liver to systemic glucose homeostasis, we wanted to investigate how exercise training influences hepatic β‐adrenergic responsiveness. We provide evidence that exercise training reduces the response of the liver to a pharmacological epinephrine challenge in vivo. In support of this, we show that in trained, compared to sedentary mice, (a) the rise in blood glucose 15 min following an epinephrine injection was significantly blunted, (b) the relative depletion in liver glycogen was reduced, (c) the phosphorylation of PKA substrates appeared to be attenuated and (d) the induction of a number of PKA‐sensitive genes was decreased. While the relative depletion in glycogen content was larger in sedentary animals, suggestive of increased glycogenolysis, both epinephrine‐treated groups displayed similar liver glycogen content. Whether the larger glucose response in sedentary mice was due to increases in the gluconeogenic pathway remains unknown. Furthermore, wheel running‐mediated adaptations in skeletal muscle and/or adipose tissue could also play a role in the reduced glucose response to an epinephrine challenge.

In addition to having direct effects on the liver, epinephrine also impacts the secretion of the pancreatic hormones such as glucagon (Hamilton et al., 2018) and insulin (Han & Bonen, 1998; Peterhoff et al., 2003). There were main effects of both exercise and epinephrine to increase circulating glucagon and reduce insulin. Taken together, these findings provide evidence that epinephrine‐induced changes in glucagon and insulin are unlikely to explain the impaired response of the liver to epinephrine in trained mice.

Epinephrine‐stimulated glycogenolysis and gluconeogenesis occurs primarily through the β‐AR coupled adenylyl cyclase (AC)‐cAMP cascade (Kawai, 1983; Schmelck & Hanoune, 1980). To determine if differences in the expression of β‐adrenergic receptors could explain the blunted response to epinephrine, we measured the mRNA expression of both α‐ and β‐adrenergic receptors. Upon epinephrine injection, we observed larger increases in hepatic *Adra1a, Adra2a,*and *Adbr1* gene expression in the sedentary compared to trained mice. This agrees with our primary observation of reduced blood glucose and liver glycogen depletion in the trained mice 15 min following epinephrine injection. While we have not been able to reliably detect the protein content of β‐adrenergic receptors by Western blotting, our gene expression analysis provides evidence that the blunted effects of epinephrine on the liver are not secondary to reductions in adrenergic receptors in trained mice.

The effects of exercise training on β‐adrenergic receptor sensitivity and density has been studied before in numerous tissues. In isolated human adipose tissue, a single bout of exercise increases catecholamine‐induced lipolysis (Crampes, Beauville, Riviere, Garrigues, & Lafontan, 1988; Riviere et al., 1989; Wahrenberg, Bolinder, & Arner, 1991; Wahrenberg, Engfeldt, Bolinder, & Arner, 1987). In myocardial membranes from swim‐trained rats, β‐adrenergic receptor number was significantly reduced in comparison to sedentary rats (Werle, Strobel, & Weicker, 1990). In trained diabetic rats, rates of sodium‐fluoride induced adenylate cyclase activity were increased in soleus but not vastus lateralis muscles, in comparison to sedentary diabetic controls (Plourde, Rousseau‐Migneron, & Nadeau, 1992). Mazzeo, Podolin, and Henry (1995) assessed β1 and β2 adrenergic receptor density and affinity in the soleus, heart, and livers from treadmill trained and sedentary rats. In the heart, β‐receptor‐binding affinity was significantly reduced in trained animals and there was a nonsignificant trend of training to reduce β1‐ and β2‐receptor density. In soleus muscle, training decreased only β1‐receptor density. However, there was no effect of exercise to alter β‐receptor density or binding affinity in the liver. In rats treated with epinephrine twice‐daily for 28 days (0.3 mg/kg), there was a marked reduction in the epinephrine‐induced glycogenolytic response in comparison to untreated control rats (Rousseau Migneron, LeBlanc, Lafrance, & Depocas, 1975). These findings are consistent with our data and would perhaps suggest that repeated surges in epinephrine with each bout of exercise could serve as a signal to reduce liver epinephrine responsiveness with training.

Activation of PDE3B or PDE4B triggers the breakdown of cAMP in the liver which can attenuate epinephrine or glucagon‐stimulated increases in cAMP and PKA signaling (Johanns et al., 2016; Xie et al., 2018). Bousquet‐Mélou et al. (1995) found that phosphodiesterase attenuated β‐adrenergic‐stimulated lipolysis in adipocytes. In our study, exercise training increased gene expression of the two main cAMP‐selective phosphodiesterases found in the liver, PDE3B and PDE4B. While there were no differences within the epinephrine‐treated groups, this suggests that exercise‐induced increases in phosphodiesterases may underlie the liver's reduced response to epinephrine. Recently, AMPK was shown to phosphorylate and activate PDE4B, and subsequently inhibit glucagon‐stimulated increases in hepatic cAMP (Johanns et al., 2016). AMPK has also been reported to regulate liver glycogenolysis during exercise (Andreelli et al., 2006; Hughey et al., 2017; Steinberg et al., 2010). However, in the current study neither exercise training nor acute treatment with epinephrine impacted liver AMPK signaling. These findings provide evidence that AMPK does not explain the attenuated responsiveness to epinephrine in livers from exercise trained mice.

Reduced hepatic β‐adrenergic responsiveness would be an advantageous adaptation in the context of exercise performance and substrate selection. It is well‐known that endurance‐trained subjects experience a reduction in glucose utilization and a consequent increase in fat oxidation during exercise, which helps to delay the onset of fatigue (Henriksson, 1977; Phillips, Tarnopolsky, & Heigenhauser, 1996). In a study by Baldwin et al. (1975), 16 weeks of swim training protected against rapid muscle and liver glycogen depletion during an acute treadmill test. The authors speculated that the reduced catecholamine levels in the trained animals may explain the glycogen sparing effect. Our data, showing an effect of training to blunt the glucose response to epinephrine, reduce glycogen depletion and the expression of adrenergic receptor genes, supports the effect of hepatic glycogen sparing and would suggest that an altered response to epinephrine could also be involved in this phenomenon.

To date, this is the first study to investigate the impact of exercise training on hepatic adrenergic responsiveness and the potential underlying mechanisms therein. Brief exercise training was able reduce the livers responsiveness to an epinephrine challenge. Exercise trained mice experienced a reduced increase in blood glucose, diminished relative depletion of liver glycogen stores, and reductions in gluconeogenic gene expression. Importantly, these effects were not recapitulated with a single, overnight bout of wheel running, suggesting that these adaptations were training dependent. From a mechanistic perspective, exercise training increased the gene expression of PDE3B and PDE4B enzymes, which might contribute to the blunted increase in PKA signaling in livers from trained mice. In the future, this will need to be tested more definitively and it will need to be determined if the observations in male mice can be recapitulated in females.

## CONFLICT OF INTEREST

The authors declare no conflicts of interest.

## AUTHOR CONTRIBUTIONS

H.A.D. and D.C.W. planned and designed the experiments. H.A.D., L.K.T., and G.L.M. conducted the experiments. H.A.D. and D.C.W analyzed data, prepared figures, and drafted the manuscript. All authors critically reviewed and approved the final version of the manuscript.
